# CYP2B6*18 is associated with nevirpine hypersensitivity independently of HLA-C*04:01 in a Malawian HIV population

**DOI:** 10.1186/2045-7022-4-S3-P126

**Published:** 2014-07-18

**Authors:** Daniel Carr, Mas Chaponda, Elena Cornejo Castro, Andrea Jorgensen, Saye Khoo, Munir Pirmohamed

**Affiliations:** 1 University of Liverpool, UK; 2 University of Liverpool, Molecular and Clinical Pharmacology, UK; 3 University of Liverpool, Biostatistics, UK; 4 University of Liverpool, Molecular and Clinical Pharamcology, UK

## 

Nevirapine (NVP), a non-nucleoside reverse transcriptase inhibitor (NNRTI) used in human immunodeficiency virus (HIV) treatment can cause hypersensitivity reactions in 6-10% of patients, which in the most serious cases (1.3%) can manifest as Stevens-Johnson Syndrome (SJS) or Toxic Epidermal Necrolysis (TEN). A total of 209 patients with NVP hypersensitivity (57 from a prospective cohort and 152 clinic patients) were compared with 463 control patients on NVP without any hypersensitivity. The case group included 70 patients with serious blistering reactions (SJS or TEN). All individuals were genotyped for 2 SNPs in the CYP2B6 gene (c.516G>T [CYP2B6*9] and c.983T>C [CYP2B6*18]) using the TaqMan real-time genotyping platform. A replication cohort of 29 controls and 31 nevirapine hypersensitive patients, including 8 SJS/TEN cases, was subsequently typed. An association between the CYP2B6*18 polymorphism and NVP-induced SJS/TEN was observed (p=0.013). In the SJS/TEN group, 30% of individuals possessed at least one *18 allele vs. 16% in the tolerant group (p=0.006; OR (95% CI) 2.24(1.27-3.94)). This association was also borderline significant in the replication cohort (p=0.075). Combined analysis resulted in an odds ratio of 2.52 (95% CI 1.48-4.20; p=0.0005) for the association of SJS/TEN with CYP2B6*18. This association was not observed for other hypersensitivity phenotypes. Data show a putative association between the CYP2B6*18 polymorphism and nevirapine-induced SJS/TEN. CYP2B6*18 has a frequency of around 5-10% in African populations but is not observed in Caucasians, and this may therefore represent an ethnicity-specific predisposing factor.

